# Optimal Dietary and Plasma Magnesium Statuses Depend on Dietary Quality for a Reduction in the Risk of All-Cause Mortality in Older Adults

**DOI:** 10.3390/nu7075244

**Published:** 2015-07-13

**Authors:** Yi-Chen Huang, Mark L. Wahlqvist, Mei-Ding Kao, Jui-Lien Wang, Meei-Shyuan Lee

**Affiliations:** 1 Graduate Institute of Life Sciences, National Defense Medical Center, 161 Minchuan East Road, Section 6, Taipei 11490, Taiwan; E-Mail: yichen711015@gmail.com; 2 Institute of Population Health Sciences, National Health Research Institutes, No 35, Keyan Road, Zhunan Town, Miaoli County 35053, Taiwan; E-Mail: profmlw@nhri.org.tw; 3 School of Public Health, National Defense Medical Center, 161 Minchuan East Road, Section 6, Taipei 11490, Taiwan; 4 Monash Asia Institute, Monash University, 5th Floor, H Building, 900 Dandenong Road, Caulfield East, Melbourne, Victoria 3145, Australia; 5 Institute of Food and Nutrition, Providence University, 200, Section 7, Taiwan Boulevard, Shalu District, Taichung City 43301, Taiwan; E-Mail: mdkao@pu.edu.tw; 6 Department of Nutrition, Master Program of Biomedical Nutrition, HungKuang University, 1018, Section 6, Taiwan Boulevard, Shalu District, Taichung City 43302, Taiwan; E-Mail: rlwang@sunrise.hk.edu.tw

**Keywords:** plasma magnesium, mortality, stroke, elderly, Nutrition and Health Survey in Taiwan (NAHSIT)

## Abstract

The association between dietary or plasma magnesium (Mg) with diabetes incidence and with mortality in free-living elderly was investigated. A total of 1400 participants from the Taiwanese Nutrition Survey, aged ≥ 65 years, and diabetes-free from the 1999–2000 were assessed. The dietary intake and plasma Mg concentration were obtained through 24h dietary recall and health examination at baseline. Participants were classified by quartiles (Q) of dietary Mg or by the plasma Mg normal range (0.75–0.95 mmol/L). Dietary diversity score (DDS, range 1–6) represented the dietary quality. During 8 and 10 years, 231 incident diabetes cases and 475 deaths were identified. Cox’s proportional-hazards regression was used to evaluate the association between Mg and health outcomes. The hazard ratios (95% confidence interval) for death in Q2 and Q3 of Mg intakes with DDS > 4 were 0.57 (0.44–0.74) and 0.59 (0.39–0.88), respectively, compared with the lowest intake and DDS ≤ 4 participants. Participants with normal and high plasma Mg in conjunction with high DDS had relative risks of 0.58 (0.37–0.89) and 0.46 (0.25–0.85) in mortality compared with low plasma Mg and lower DDS. Optimal dietary Mg intake and plasma Mg depend on dietary quality to reduce the mortality risk in older adults.

## 1. Introduction

Magnesium (Mg) is a major intracellular cation and an essential mineral. It is crucial for the maintenance of bone health and in calcium and potassium absorption [1,2]. Several studies have demonstrated that Mg is inversely associated with the risk of chronic disease, as reflected in inflammatory biomarkers [3], coronary heart disease [4], diabetes [5], and stroke [6]. Moreover, aging with Mg deficits favors oxygen-derived free radical formation and low-grade inflammation and may lead to frailty, to which sarcopenia may contribute [7]. Sarcopenia is highly correlated with the risk of mortality in later life [8]. 

The intake of Mg does not meet the Dietary Reference Intakes (DRIs) for Taiwanese elderly people [9]. Older people may have inadequate Mg intakes for several reasons. First, the capacity of the skeleton to store and release Mg decreases with age [7]. Second, renal Mg excretion increases with age [7]. Third, the intake of Mg-dense foods and the likely bioavailability of these foods diminishes with age. For example, appetite and taste are compromised with age, chewing difficulties increase, and medication use is frequently inadequate [10]. Mg-rich foods are mostly unprocessed foods such as whole grains, vegetables (particularly green leafy and beans), fruits, and nuts. These foods also provide other associated essential nutrients including dietary fiber [2]. However, older adults tend to consume monotonous diets, processed foods, and fewer whole grain products because of chewing difficulty [11]. The resultant limited dietary quality is inversely associated with increased mortality in older people [12]. Whether dietary quality modifies any association between Mg status and mortality is not known, particularly in vulnerable populations such as older people.

Taiwan is a country in Asia with a relatively low incidence of obesity, diabetes, and cardiovascular disease (CVD) and a unique dietary pattern when compared to Western countries [13]. Evidence for an association between Mg status and the risk of diabetes, or of increased mortality, based on representative population-based dietary and laboratory data in Asian countries, is limited. Dietary quality may be a key factor in whether a perceived association exists between Mg status and cause-specific or all-cause mortality in Asia among elderly people. There has been interest in the roles that Mg deficiency might have in both the pathogenesis of diabetes and in cardiac death related to arrhythmia [14]. It is conceivable that underlying Mg deficiency might account for both diabetes (and pre-diabetes) and, in part, for cardiac mortality. The present study investigated whether dietary Mg intake or plasma Mg in conjunction with dietary quality accounts for the risk of diabetes and all-cause and cause-specific mortality among representative free-living community-based older Taiwanese people.

## 2. Materials and Methods

### 2.1. Study Population 

All participants were recruited from the representative Nutrition and Health Survey in Taiwan (NAHSIT), conducted in 1999–2000. A total of 1937 elderly people aged 65 years or older completed a household interview. Sixty-four participants who had an incorrect date of interview or did not have a personal ID were excluded. Furthermore, 150 participants were excluded who provided implausible total daily energy intakes (for men <800 kcal or >4200 kcal and for women <500 kcal or >3500 kcal), and 323 participants were excluded who were previously diagnosed as having diabetes before the interview. Therefore, 1400 and 986 participants were eligible for the dietary Mg and plasma Mg studies, respectively. All information, including baseline characteristics, dietary information, medical history, and personal behaviors, was collected through a face-to-face interview. More detailed information about NAHSIT is provided elsewhere [15]. The study was approved by the Ethical Review Boards of Academic Sinica and the National Health Research Institutes in Taiwan.

### 2.2. Dietary Information

Dietary information was collected through a 24h dietary recall and a validated simplified food frequency questionnaire (SFFQ) for the previous month to calculate nutrient and specific food group intakes, respectively. From the 24h dietary recall, the total Mg intake was calculated using the Taiwanese food composition data [16]. The intra-class correlation coefficient (ICC) for Mg for three repeated 24h dietary recalls was 0.65. The ICCs for energy and other nutrients ranged from 0.13 to 0.84 with an average of 0.48 (data not shown). The methodological details are reported elsewhere [17]. Participants were divided into four groups according to quartiles (Q) of dietary Mg intake; the cut-off points being <155, 155–<205, 205–<265, and ≥265 mg/day. The dietary diversity score (DDS) (range 1–6) was used to assess dietary quality and comprised six food groups, including grains, dairy products, vegetables, fruits, and cooking oil as well as meat/fish/eggs/soy [12]. At least a half a serving per day of each food group from the 24h recall was required for a DDS score of 1. Gender-specific serving sizes and Mg content for each food group obtained from the 24h recalls were used together with the SFFQ to generate usual daily food group intake as servings and the population-weighted mean of Mg intake [12]. This enabled us to increase the level of confidence placed in food group source of Mg and validate the result for the Mg intake from the 24h dietary recall. We have shown that the Spearman rank correlation coefficients between frequencies of food group obtained from the SFFQ and from the average of multiple 24h dietary recalls ranged from 0.132 to 0.678 for men and 0.052 to 0.759 for women [18].

### 2.3. Plasma Magnesium and Other Biomarkers

Plasma Mg concentration was measured by a colorimetric method using an Olympus Autoanalyzer. A plasma Mg concentration of 0.75 to 0.95 mmol/L (1.8 to 2.3 mg/dL) was used as the normal range for clinical diagnostic purposes [1]. Therefore, three groups were defined as follows: low (<0.75 mmol/L), normal (0.75–0.95 mmol/L), and high (>0.95 mmol/L). The plasma glucose, serum C-reactive protein (CRP), and serum creatinine concentrations were also measured at baseline to establish histories of diabetes, inflammatory disorders, and impaired kidney function.

### 2.4. Outcome Ascertainment

The NAHSIT 1999–2000 data set was linked to the National Health Insurance and National Death Registry data to determine incident diabetes and survival from the date of interview to 31 December 2006 and 31 December 2008, respectively. Disease and cause-specific death were defined based on the International Classification of Disease, Ninth Revision, Clinical Modification (ICD-9-CM) and Taiwanese A-code (pre-ICD). Patients with diabetes were defined as those who had at least two records of diabetes, codes A181 (before 2000) or ICD-CM code 250, within one year between 1999 and 2006. Cause of death from CVD was defined as ICD-9 390–459. 

### 2.5. Statistical Analysis

All statistical analyses were performed using SAS 9.2 and SUDAAN 9.0. Continuous and nominal data were expressed as mean ± standard error (SE) and percentage, respectively. Chi-square and analysis of variance (ANOVA) were used to compare the differences between baseline characteristics, food and nutrient intakes, and Mg statuses. All nutrient intakes were adjusted for energy intake. Time to follow-up was calculated from baseline, 1999, to 31 December 2006 for incident diabetes cases and to 31 December 2008 for death. Cox proportional-hazard regression was used to calculate the hazard ratio (HR) and 95% confidence interval (CI) to evaluate the associations between dietary Mg intake or plasma Mg and the risks of diabetes or mortality.

Models were adjusted for confounding factors. In the dietary analyses, adjustments were made for age, gender, education level (illiterate, primary school, high school, and above), personal income (<5000, 5000–19,999, ≥20,000 NT$/month), smoking more than six months (no, former smoker, current smoker), drinking (never drank, former drinker, current drinker), physical activity (<1.5, 1.5–2.9, ≥3 metabolic equivalent (MET)/day), body mass index (BMI) (<18.5, 18.5–23.9, 24.0–26.9, ≥27.0), appetite (good, fair, and poor), total energy intake, and dietary fiber intake. Missing values for each variable were assigned to a discrete group for model adjustment. For plasma Mg analyses, adjustments were made for age, gender, education level, physical activity, BMI, dietary fiber, and serum creatinine concentration. Tests for trend were conducted for dietary and plasma Mg regression models as continuous variables. For the joint effect analyses, the cut-off point for the DDS score was 4 to determine the associations with high and low dietary quality.

Extensive sensitivity analyses were undertaken to consider preclinical outcomes (by exclusion of first year cases), renal function (by exclusion of creatinine >1.5 mg/dL) [19], and smoking. Further adjustments were made for chronic disease history, and dietary factors.

## 3. Results

The median durations of follow-up were 6.74 and 8.73 years for diabetes incidence and survivorship, respectively. The baseline characteristics of participants according to quartiles of dietary Mg intake are shown in Table 1. The participants in the highest group of dietary Mg were likely to be more educated, have more personal income, be non-smokers, be former drinkers, have satisfactory chewing ability and a good appetite, be less underweight, have a higher BMI, and be more physically active (*p* < 0.05). The dietary diversity scores, total energy intake, and wholegrain, dairy product, fruit, egg, cholesterol, dietary fiber, potassium, and calcium intakes were greater with increasing dietary Mg intake (all *p* < 0.05). However, the fat and saturated fatty acid intakes were lower with lower dietary Mg intake.

**Table 1:** Distributions of demographics and study variables by magnesium (Mg) intake in NAHSIT 1999–2000 participants ^†^ (*n* = 1400).

Descriptor	Total	Mean ± SE	Mg intake (mg/day)				
			Q1 (<155)	Q2 (155–<205)	Q3 (205–<265)	Q4 (≥265)	p ‡
Total, *n*			401	336	330	333	
Median			126	178	230	325	
Plasma Mg (mmol/L), mean ± SE	0.91 ± 0.01		0.90 ± 0.01	0.90 ± 0.01	0.92 ± 0.01	0.91 ± 0.01	<0.05
Gender, %							0.14
Men	52.5	226 ± 5.99	45.4	59.1	53.6	52.1	
Women	47.5	223 ± 7.44	54.7	40.9	46.4	47.9	
Age at baseline, %							<0.05
65–69	30.0	228 ± 5.32	26.1	31.9	34.8	27.4	
70–74	30.5	227 ± 7.92	27.9	32.9	28.1	33.1	
75–97	39.5	220 ± 9.14	46.0	35.2	37.1	39.5	
Education, %							<0.01
Illiterate	37.5	212 ± 7.55	48.7	33.1	35.9	32.0	
Primary school	39.7	218 ± 6.96	40.7	46.4	38.5	33.3	
High school and above	22.8	258 ± 9.00	10.5	20.5	25.6	34.8	
Ethnicity, %							0.24
Non indigenous	98.2	225 ± 6.03	97.2	98.4	98.2	99.0	
Indigenous	1.81	194 ± 14.6	2.78	1.63	1.79	1.01	
Live alone, %							0.75
No	88.9	232 ± 7.31	88.2	91.2	88.6	87.7	
Yes	11.1	229 ± 10.6	11.8	8.83	11.4	12.3	
Personal income (NTD/month), %							<0.01
<5000	62.2	219 ± 7.20	69.2	57.7	65.1	56.7	
5000–19,999	28.4	224 ± 6.57	26.7	35.5	22.9	28.4	
≥20,000	9.42	261 ± 14.2	4.06	6.83	12.1	14.9	
Smoking more than 6 months, %							<0.01
No	63.1	230 ± 7.21	64.0	54.4	62.7	71.0	
Former smoker	14.7	233 ± 10.3	11.7	16.5	15.0	15.7	
Current smoker	22.2	204 ± 7.24	24.3	29.1	22.3	13.3	
Alcohol drinker, %							0.01
Never drinker	74.4	221 ± 6.39	82.0	70.8	70.3	74.6	
Former drinker	7.10	243 ± 17.6	5.18	7.59	6.92	8.73	
Current drinker	18.5	233 ± 8.16	12.8	21.6	22.8	16.7	
Chewing ability, %							<0.01
Satisfactory	65.3	230 ± 5.80	58.8	68.8	61.2	72.5	
Unsatisfactory	34.7	216 ± 8.62	41.2	31.2	38.8	24.5	
Appetite, %							<0.01
Good	34.9	241 ± 9.44	23.1	33.9	41.0	41.6	
Fair	58.3	216 ± 5.65	65.6	60.5	55.1	51.8	
Poor	6.80	213 ± 15.8	11.2	5.58	3.90	6.56	
Body mass index (kg/m^2^), %							0.08
<18.5	7.00	225 ± 14.4	8.67	6.56	4.24	8.52	
18.5–23.9	49.6	222 ± 9.12	50.5	51.3	50.9	46.0	
24.0–26.9	27.9	234 ± 9.99	27.0	29.4	25.3	29.7	
≥27.0	15.5	235 ± 10.5	13.8	12.8	19.6	15.8	
MET (/day), %							<0.01
<1.5	56.4	213 ± 7.35	70.6	56.2	48.7	50.0	
1.5–2.9	10.8	226 ± 12.3	10.3	10.1	12.1	10.7	
≥3	32.8	244 ± 5.96	19.2	33.7	39.2	39.3	
History of hypertension, %							0.57
No	70.7	222 ± 6.13	69.7	73.7	69.3	70.0	
Yes	29.3	229 ± 10.3	30.3	26.3	30.7	30.0	
Hyperglycemic, %							0.55
No (<126 mg/dL)	91.3	229 ± 6.19	88.5	92.7	92.2	91.8	
Yes (≥126 mg/dL)	8.70	224 ± 16.2	11.5	7.32	7.78	8.25	
Incident diabetes, %							0.56
No	83.2	226 ± 6.11	80.9	83.7	81.2	86.9	
Yes	16.8	218 ± 11.1	19.1	16.3	18.8	13.1	
Plasma glucose (mg/dL), mean ± SE	106 ± 1.25		111 ± 4.23	103 ± 1.34	104 ± 0.91	108 ± 2.37	0.24
Serum C-reactive protein, mean ± SE	0.45 ± 0.04		0.42 ± 0.05	0.43 ± 0.05	0.44 ± 0.04	0.51 ± 0.14	0.93
Serum creatinine (mg/dL), mean ± SE	1.01 ± 0.02		1.03 ± 0.06	0.96 ± 0.02	1.03 ± 0.03	1.00 ± 0.03	0.03
Serum creatinine >1.5 mg/dL, %	5.85		9.12	3.26	7.01	4.17	0.09
Dietary diversity score, mean ± SE	4.53 ± 0.06		3.89 ± 0.08 ^abc^	4.50 ± 0.08 ^ad^	4.74 ± 0.07 ^b^	5.00 ± 0.09 ^cd^	<0.01
≤4, %	46.3	194 ± 5.44	74.5	46.7	36.8	27.0	
>4, %	53.7	252 ± 7.97	25.6	53.3	63.3	73.0	
Dietary intake from SFFQ (serving/day), mean ± SE							
Total grain	11.3 ± 0.22		11.1 ± 0.35	11.8 ± 0.45	11.0 ± 0.30	11.3 ± 0.27	0.45
Whole grain	0.30±0.07		0.06 ± 0.03 ^a^	0.19 ± 0.06 ^b^	0.20 ± 0.09 ^c^	0.73 ± 0.17 ^abc^	<0.01
Dairy product	0.84 ± 0.05		0.58 ± 0.07 ^ab^	0.76 ± 0.07^c^	0.98 ± 0.08 ^a^	1.04 ± 0.07 ^bc^	<0.01
Vegetable	2.55 ± 0.11		2.36 ± 0.16	2.51 ± 0.17	2.57 ± 0.10	2.74 ± 0.13	0.07
Fruit	1.30 ± 0.04		1.00 ± 0.08 ^abc^	1.28 ± 0.07 ^ad^	1.36 ± 0.07 ^b^	1.56 ± 0.07 ^cd^	<0.01
Egg	0.23 ± 0.01		0.17 ± 0.02 ^a^	0.23 ± 0.03	0.23 ± 0.01	0.29 ± 0.02 ^a^	<0.01
Dietary information from 24 h recall, mean ± SE							
Total energy intake (kcal/day)	1633 ± 42.1		1490 ± 55.6 ^a^	1616 ± 57.1	1664 ± 59.1	1763 ± 59.0 ^a^	<0.01
Nutrient intakes per 1000 kcal							
Fat (g)	28.7 ± 0.53		32.9 ± 1.26 ^abc^	27.7 ± 1.11 ^a^	27.6 ± 0.91 ^b^	26.5 ± 0.74 ^c^	<0.01
Saturated fatty acid (mg)	8665 ± 213		10,175 ± 448^a^	8472 ± 383	8494 ± 429	7491 ± 297^a^	<0.01
Cholesterol (mg)	129 ± 4.76		112 ± 6.59 ^ab^	133 ± 9.43	137 ± 6.70 ^a^	136 ± 7.70 ^b^	0.02
Dietary fiber (g)	12.5 ± 0.45		8.55 ± 0.32 ^abc^	10.8 ± 0.44 ^ade^	13.2 ± 0.66 ^bdf^	17.4 ± 0.91 ^cef^	<0.01
Potassium (mg)	1483 ± 34.9		932 ± 29.2 ^abc^	1243 ± 36.0 ^ade^	1634 ± 60.0 ^bdf^	2132 ± 52.7 ^cef^	<0.01
Calcium (mg)	411 ± 14.9		208 ± 12.1 ^abc^	328 ± 14.9 ^ade^	456 ± 19.0 ^bdf^	655 ± 24.9 ^cef^	<0.01

^†^ Total sample size is 1400; cases with missing values were not included for the relevant variable. **^‡^** ANOVA and chi-square were used for continuous and categories variables by SUDAAN program. Where there is the same superscript it means that there is significant difference between the two groups by Bonferroni test. Percentages are weighted to reflect their representativeness in the population. MET: metabolic equivalent; SFFQ: simplified food frequency questionnaire.

Table 2 shows the HRs (95% CI) of dietary Mg for risk of diabetes as well as all-cause and disease-specific mortalities. The median intakes of Mg were 206 mg/day and 200 mg/day for non-diabetes and diabetes, respectively, in our cohort. The HRs (95% CI) for incident diabetes in Q2 to Q4 of Mg intake were 0.75 (0.42–1.32), 0.88 (0.60–1.30), 0.61 (0.31–1.19) compared with the lowest intake group (Q1) in the crude model. All HRs remained non-significant after full adjustment for covariates in Model 3.

**Table 2:** Hazard ratio of magnesium (Mg) intake for risk of all-cause mortality and incidence of diabetes in NAHSIT elderly.

	Mg Intake (mg/day) Hazard Ratio (95% Confidence Intervals)				
	Q1 (<155)	Q2 (155–< 205)	Q3 (205–< 265)	Q4 (≥265)	*p* for trend
**Diabetes incidence**					
Person-years	2096.5	1948.3	1885.1	1964.0	
No of events	71	53	64	43	
Cumulative incidence rate/1000 person-years	33.9	27.2	34.0	21.9	
Crude model	1.00	0.75 (0.42–1.32)	0.88 (0.60–1.30)	0.61 (0.31–1.19)	0.14
Model 1	1.00	0.77 (0.42–1.40)	0.85 (0.57–1.27)	0.57 (0.27–1.20)	0.11
Model 2	1.00	0.74 (0.42–1.32)	0.82 (0.56–1.19)	0.54 (0.26–1.13)	0.08
Model 3	1.00	0.77 (0.43–1.38)	0.86 (0.57–1.31)	0.59 (0.26–1.33)	0.20
**All-cause mortality**					
Person-years	2795.7	2556.7	2568.8	2565.2	
No of events/survivals	173	112	93	97	
Cumulative death rate/1000 person-years	61.9	43.8	36.2	37.8	
Crude model	1.00	0.65 (0.48–0.87)	0.57 (0.42–0.78)	0.64 (0.45–0.90)	0.01
Model 1	1.00	0.71 (0.52–0.97)	0.68 (0.50–0.93)	0.85 (0.60–1.19)	0.22
Model 2	1.00	0.75 (0.55–1.03)	0.75 (0.56–1.00)	0.89 (0.62–1.27)	0.42
Model 3	1.00	0.79 (0.58–1.08)	0.81 (0.60–1.09)	1.05 (0.74–1.49)	0.94
**Cardiovascular mortality**					
Person-years	2223.0	2173.7	2223.5	2227.2	
No of events/survivals	40	33	27	24	
Cumulative death rate/1000 person-years	18.0	15.2	12.1	10.8	
Crude model	1.00	1.04 (0.52–2.09)	0.77 (0.42–1.43)	0.77 (0.42–1.40)	0.26
Model 1	1.00	1.10 (0.64–1.90)	1.02 (0.52–2.00)	1.08 (0.54–2.17)	0.89
Model 2	1.00	1.23 (0.71–2.13)	1.16 (0.63–2.14)	1.14 (0.55–2.36)	0.75
Model 3	1.00	1.25 (0.71–2.18)	1.21 (0.60–2.45)	1.24 (0.59–2.60)	0.61

Cox’s proportional hazard models were used to estimate HR with the SUDAAN program. Model 1: adjusted for age (cont.), gender, education level, personal income, smoking, drinking, physical activity, BMI; Model 2: adjusted for covariates in model 1 plus appetite; Model 3: adjusted for covariates in model 2 plus dietary fiber and energy intake.

The median intakes of Mg were 212 mg/day and 190 mg/day for the survivors and deceased, respectively, in our cohort. However, those participants with higher Mg intakes between Q2 and Q4 had a lower risk of all-cause mortality, with HRs (95% CI) 0.65 (0.48–0.87), 0.57 (0.42–0.78) and 0.64 (0.45–0.90), respectively, compared with the lowest group (Q1) in the crude model (*p* for trend = 0.01). Again, the HRs (95% CI) were 0.71 (0.52–0.97), 0.68 (0.50–0.93), and 0.85 (0.60–1.19) in Model 1 (*p* for trend = 0.22), after adjustment for confounding variables. With further adjustment for appetite and dietary variables, the significance of HRs disappeared in Model 2 and Model 3. The CVD is the top 2 cause-specific mortalities of this study population (Appendix Table A1). CVD cause-specific mortality was not significant (HR = 1.24; 95% CI = 0.59–2.60 in Q4). 

**Figure 1:**
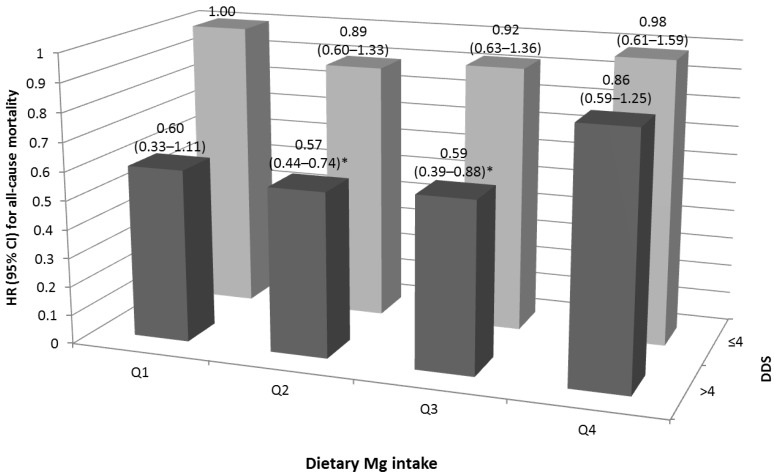
Joint hazards ratios for all-cause mortality in older Taiwanese in regard to dietary Mg intake and dietary diversity score (adjusted for age, gender, education level, personal income, smoking, drinking, body mass index, physical activity, appetite, dietary fiber and total energy intake). * *p* < 0.05.

Since there was a significant interaction between dietary Mg intake and DDS for all-cause mortality (*p* = 0.005), joint analyses were performed (Figure 1). Those with a dietary Mg intake in Q2 or Q3 and a higher DDS (>4) had a 43% and 41% lower risk of mortality, respectively, compared with participants with the lowest Mg intake and DDS (≤4); the HRs (95% CI) were 0.57 (0.44–0.74) and 0.59 (0.39–0.88) in the fully adjusted model.

When Mg intakes were derived from the SFFQ, rather than the 24h recall, the findings were similar. The joint effect with DDS on all-cause mortality was again seen, although in the Q1 (HR = 0.53, 95% CI = 0.37–0.77) and Q2 (HR = 0.65, 95% CI = 0.45–0.95) intakes rather than the Q2 and Q3 (data not shown).

The baseline characteristics of the plasma Mg status are shown in Table 3. Participants in the highest group for plasma Mg had higher education levels, were less likely to be indigenous people, and had higher serum creatinine concentrations (*p* < 0.05). When the DDS, vegetable, egg, and calcium intakes were greater, plasma Mg was higher, particularly with increments in DDS and eggs.

**Table 3:** Distributions of demographics and study variables by plasma magnesium (Mg) status in NAHSIT 1999–2000 participants ^†^ (*n* = 986).

Descriptor	Total	Plasma Mg (mmol/L)				*p* ^‡^
		Mean ± SE	Low (<0.75)	Normal (0.75–0.95)	High (>0.95)	
Total, *n*			44	752	190	
Median			0.74	0.90	0.99	
Mg intake (mg/day), mean ± SE	227 ± 7.22		217 ± 18.4	223 ± 7.84	241 ± 11.0	0.28
Gender, %						0.24
Men	53.5	0.91 ± 0.01	64.3	52.2	57.1	
Women	46.5	0.91 ± 0.01	35.7	47.8	42.9	
Age at baseline, %						0.56
65–69	32.4	0.90 ± 0.00	27.7	33.2	30.1	
70–74	30.9	0.90 ± 0.01	28.4	31.6	28.8	
75–97	36.7	0.91 ± 0.01	43.9	35.3	41.1	
Education, %						0.03
Illiterate	35.8	0.91 ± 0.01	40.6	34.7	39.0	
Primary school	41.9	0.90 ± 0.01	50.5	43.8	34.0	
High school and above	22.3	0.92 ± 0.01	8.91	21.5	27.0	
Ethnicity, %						<0.01
Non indigenous	97.8	0.91 ± 0.01	84.8	97.8	99.1	
Indigenous	2.25	0.84 ± 0.02	15.2	2.21	0.95	
Live alone, %						0.66
No	88.9	0.91 ± 0.01	85.9	88.4	91.1	
Yes	11.1	0.90 ± 0.02	14.1	11.6	8.86	
Personal income (NTD/month), %						0.06
<5000	62.8	0.91 ± 0.01	69.5	63.1	61.2	
5000–19,999	26.1	0.90 ± 0.01	30.5	26.5	24.3	
≥20,000	11.1	0.93 ± 0.01	--	10.4	14.4	
Smoking more than 6 months, %						0.55
No	62.2	0.91 ± 0.0	59.7	63.7	57.1	
Former smoker	14.8	0.91 ± 0.01	8.71	14.3	17.6	
Current smoker	23.0	0.90 ± 0.01	31.6	22.1	25.3	
Alcohol drinker, %						0.12
Never drinker	73.7	0.90 ± 0.01	77.8	74.7	69.6	
Former drinker	7.54	0.92 ± 0.01	1.39	7.15	9.67	
Current drinker	18.8	0.91 ± 0.01	20.8	18.2	20.8	
Chewing ability, %						0.72
Satisfactory	66.2	0.91±0.01	58.1	66.1	67.2	
Unsatisfactory	33.9	0.90±0.01	41.9	33.9	32.8	
Appetite, %						0.90
Good	35.9	0.90 ± 0.01	35.6	36.8	33.0	
Fair	58.3	0.91 ± 0.01	57.5	57.6	61.0	
Poor	5.78	0.91 ± 0.01	6.87	5.69	5.99	
Body mass index (kg/m^2^), %						0.97
<18.5	6.26	0.90 ± 0.01	9.76	6.32	5.62	
18.5–23.9	49.5	0.90 ± 0.01	50.4	49.8	48.1	
24.0–26.9	28.0	0.91 ± 0.01	29.1	28.0	28.3	
≥27.0	16.2	0.91 ± 0.01	10.7	15.9	18.0	
MET (/day), %						0.84
<1.5	56.0	0.90 ± 0.01	57.1	56.7	53.1	
1.5–2.9	10.3	0.90 ± 0.01	14.5	10.1	10.4	
≥3	33.8	0.91 ± 0.01	28.4	33.2	36.5	
History of hypertension, %						0.13
No	72.3	0.90 ± 0.01	69.4	73.9	66.6	
Yes	27.7	0.92 ± 0.01	30.6	26.1	33.5	
Hyperglycemic, %						0.19
No (<126 mg/dL)	91.9	0.88 ± 0.01	81.7	91.5	94.3	
Yes (≥126 mg/dL)	8.14	0.91 ± 0.01	18.3	8.49	5.71	
Incident diabetes, %						0.75
No	84.2	0.91 ± 0.01	78.6	84.0	85.6	
Yes	15.8	0.90 ± 0.01	21.4	16.0	14.4	
Plasma glucose (mg/dL), mean ± SE	106 ± 1.00		118 ± 13.6	106 ± 1.04	104 ± 2.16	0.53
Serum C-reactive protein, mean ± SE	0.42 ± 0.03		0.73 ± 0.26	0.42 ± 0.04	0.38 ± 0.04	0.42
Serum creatinine (mg/dL), mean ± SE	1.00 ± 0.02		1.01± 0.05	0.97 ± 0.02 ^a^	1.10 ± 0.04 ^a^	0.02
Serum creatinine > 1.5 mg/dL, %	5.75		0.94	4.35	11.5	0.01
Dietary diversity score, mean ± SE	4.60 ± 0.06		3.88 ± 0.19 ^ab^	4.57 ± 0.06 ^ac^	4.82 ± 0.09 ^bc^	<0.01
≤4, %	44.0	0.89 ± 0.01	77.5	46.9	29.9	<0.01
> 4, %	56.0	0.92 ± 0.01	22.5	53.1	70.1	
Dietary intake from SFFQ (serving/day), mean ± SE						
Total grain	11.6 ± 0.19		11.1 ± 0.96	11.7 ± 0.20	11.3 ± 0.34	0.38
Whole grain	0.35 ± 0.09		0.10 ± 0.10	0.31 ± 0.09	0.55 ± 0.18	0.15
Dairy product	0.84 ± 0.06		0.52 ± 0.18	0.83 ± 0.08	0.90 ± 0.07	0.17
Vegetable	2.64 ± 0.12		2.08 ± 0.22 ^a^	2.61 ± 0.12	2.80 ± 0.16 ^a^	<0.05
Fruit	1.33 ± 0.06		0.95 ± 0.20	1.34 ± 0.07	1.34 ± 0.08	0.16
Egg	0.23 ± 0.02		0.12 ± 0.03 ^ab^	0.23 ± 0.02 ^a^	0.26 ± 0.02 ^b^	<0.01
Dietary information from 24 h recall, mean ± SE						
Total energy intake (kcal/day)	1659 ± 50.3		1705 ± 132	1637 ± 47.9	1733 ± 90.3	0.38
Nutrient intakes per 1000 kcal						
Fat (g)	28.7 ± 0.66		25.7 ± 2.35	29.4 ± 0.79 ^a^	26.5 ± 0.66 ^a^	<0.01
Saturated fatty acid (mg)	8666 ± 243		7202 ± 962	8850 ± 276	8148 ± 373	0.04
Cholesterol (mg)	130 ± 5.66		139 ± 17.9	130 ± 5.95	125 ± 11.0	0.82
Dietary fiber (g)	12.7 ± 0.57		11.0 ± 1.43	12.4 ± 0.70	13.8 ± 0.71	0.24
Potassium (mg)	1498 ± 46.4		1297 ± 151	1462 ± 42.0	1656 ± 89.4	0.04
Calcium (mg)	411 ± 18.1		277 ± 39.7 ^ab^	402 ± 15.2 ^a^	462 ± 48.6 ^b^	0.01

^†^ Total sample size is 986; cases with missing values were not included for the relevant variable. ^‡^ ANOVA and chi-square were used for continuous and categories variables by SUDAAN program. Where there is the same superscript it means that there is significant difference between the two groups by Bonferroni test. Percentages are weighted to reflect their representativeness in the population. MET: metabolic equivalent; SFFQ: simplified food frequency questionnaire.

The HRs (95% CI) for risk of incident diabetes as well as all-cause and CVD death by plasma Mg are shown in Table 4. The HRs (95% CI) for incident diabetes were 2.05 (0.68–6.15) and 0.91 (0.58–1.42) for participants in the low and high groups of plasma Mg compared to the normal group in the fully adjusted Model 3. For all-cause mortality, the HRs (95% CI) were 1.53 (0.89–2.64) and 0.96 (0.70–1.32) in the crude model. Further adjustment for dietary fiber intake as an index of plant food intake exerted little effect on HRs. Notably, given that plasma Mg is higher when the serum creatinine is higher, the HR in the high group for plasma Mg (95% CI) was 0.70 (0.50–0.98) compared to the normal group after adjustment for serum creatinine level in Model 3 (*p* for trend = 0.02). For CVD mortality, the HRs (95% CI) for high and low plasma Mg groups were 2.28 (0.90–5.75) and 0.51 (0.20–1.26) in the fully adjusted model (Model 3), and the *p* for trend was borderline significant.

**Table 4:** Hazard ratio of plasma magnesium (Mg) for risk of all-cause and incidence of diabetes in NAHSIT elderly.

	Plasma Mg (mmol/L) Hazard Ratio (95% Confidence Intervals)			
	Low (<0.75)	Normal (0.75–0.95)	High (>0.95)	*p* for trend
**Incident diabetes**				
Person-years	198.8	4412.4	1092.5	
No of events	8	117	31	
Cumulative incidence rate/1000 person-years	40.2	26.5	28.4	
Crude model	1.63 (0.56–4.74)	1.00	0.91 (0.58–1.40)	0.45
Model 1	2.00 (0.66–6.08)	1.00	0.95 (0.61–1.47)	0.44
Model 2	2.01 (0.67–6.05)	1.00	0.96 (0.61–1.51)	0.44
Model 3	2.05 (0.68–6.15)	1.00	0.91 (0.58–1.42)	0.39
**All-cause mortality**				
Person-years	275.2	5779.6	1468.6	
No of events	23	230	54	
Cumulative incidence rate/1000 person-years	83.6	39.8	36.8	
Crude model	1.53 (0.89–2.64)	1.00	0.96 (0.70–1.32)	0.49
Model 1	1.25 (0.83–1.86)	1.00	0.86 (0.61–1.20)	0.23
Model 2	1.24 (0.83–1.86)	1.00	0.86 (0.61–1.21)	0.24
Model 3	1.24 (0.85–1.81)	1.00	0.70 (0.50–0.98)	0.02
**Cardiovascular mortality**				
Person-years	219.1	4958.0	1269.6	
No of events	8	63	13	
Cumulative incidence rate/1000 person-years	36.5	12.7	10.2	
Crude model	2.76 (0.98–7.79)	1.00	0.84 (0.40–1.75)	0.37
Model 1	2.24 (0.88–5.73)	1.00	0.76 (0.36–1.60)	0.25
Model 2	2.24 (0.88–5.74)	1.00	0.75 (0.36–1.59)	0.25
Model 3	2.28 (0.90–5.75)	1.00	0.51 (0.20–1.26)	<0.05

Cox’s proportional hazard models were used to estimate HR with the SUDAAN program. Model 1: adjusted for age (cont.), gender, education level, ethnicity, physical activity, BMI; Model 2: adjusted for covariates in model 1 plus dietary fiber; Model 3: adjusted for covariates in model 2 plus serum creatinine.

**Figure 2:**
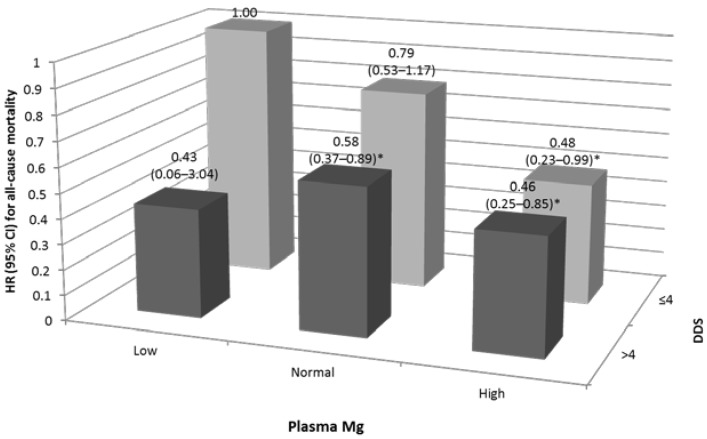
Joint hazards ratios for all-cause mortality in older Taiwanese in regard to plasma Mg and dietary diversity score (adjusted for age, gender, education level, ethnicity, body mass index, physical activity, dietary fiber and serum creatinine) * *p* < 0.05.

The interaction between plasma Mg and DDS for all-cause mortality was significant (*p* = 0.03). The joint analyses for plasma Mg concentration and DDS are shown in Figure 2. Participants with a high DDS (>4) had a lower risk of all-cause mortality; the HRs (95% CI) for normal and high plasma Mg concentrations were 0.58 (0.37–0.89) and 0.46 (0.25–0.85), respectively, compared with those with both low DDS and plasma Mg after adjustment for confounding factors. 

## 4. Discussion

### 4.1. Main Findings

In this representative prospective cohort for older adults, we found that plasma Mg concentration, but not dietary Mg intake, was inversely related to all-cause mortality risk. However, a joint effect of optimal dietary Mg and high plasma Mg with DDS in relation to all-cause mortality was observed in this relatively low dietary Mg intake population during follow-ups after a median of 8.73 years. The median Mg intake among the participants was 205 mg/day compared with 326 mg/day for Caucasian men in the U.S. National Health and Nutrition Examination Survey [20].

### 4.2. Mg and Diabetes Incidence

The risk of hypomagnesemia increases in type 2 diabetes patients [21]. However, Mg deficiency can also lead to an increased risk of type 2 diabetes [22]. Higher Mg intakes are inversely associated with incident diabetes [5] and the metabolic syndrome [3]. A longitudinal study in Taiwan (the Cardiovascular Disease Risk Factor Two-township cohort), found that a lower Mg intake (<241 mg/day) increased the risk of diabetes 1.61-fold during a 4.6 year follow-up [23]. However, we did not find an association between dietary or plasma Mg and the risk of incident diabetes in this study. The mean age of type 2 diabetes diagnosis in Taiwan was 58.7 years from 1999 to 2004 with a secular trend towards an increased incidence among younger adults, making the association among diabetes incidence and its putative risk factors such as Mg deficiency with age difficult to assess [24]. However, we know that new-onset diabetes in older adults is related to their poor dietary quality and physical inactivity as middle-aged adults [25]. Mg status in younger adults might determine this risk of diabetes in older adults. In addition, the extent and severity of comorbidities of diabetes in later life are likely to reflect earlier dietary quality and its associated nutrient densities [26].

### 4.3. Mg and Mortality

A Spanish prospective study found that adults at high CVD risk who had the highest Mg intake (median > 454 mg/day) were at a 37% lower risk of all-cause mortality [27]. In the present study, even though those in the highest Mg intake group (median 325 mg/day) had a 36% lower death risk in the crude model, this was not evident in the fully adjusted model. Older adults have a lower Mg status partly because of lower food intake, but also because Mg absorption from the gut decreases, and renal Mg excretion increases with age and medication usage [7]. Further compounding the risk of Mg deficiency, its features may include loss of appetite, nausea, vomiting, and weakness. By way of illustration, we found that those with the lowest Mg intake had less satisfactory chewing ability and appetite. These observations underscored the likelihood of poor dietary quality and associated marginal Mg intakes that are observed among older people. However, from our study, we were unable to conclude that we identified a causal relationship between diet and Mg status because both were ascertained at baseline. Moreover, the dietary methodology used did not necessarily reflect long-term dietary habits or trends.

In the population-based Study of Health in Pomerania with a median follow-up of 10.1 years, a low serum Mg concentration (≤0.73 mmol/L) was found to be associated with a higher risk of all-cause and CVD mortality [28]. In our study, we found that high plasma Mg (>0.95) was associated with a 29% lower risk of all-cause, but not CVD, mortality. The differences between the studies may be attributable to the broader contextual differences in risk populations, dietary methodologies, and food compositional databases.

Blood Mg concentration is homeostatically regulated, and extracellular Mg represents only 1% of total body Mg [29]. Mg absorption is approximately 40%–60% of intake in healthy older adults under controlled dietary conditions [30]. However, total body and extracellular Mg concentration is affected by age-related diseases, notably those affecting appetite, food intake, and renal function as well as various therapeutic agents such as diuretics [7]. In our study, the Spearman correlation coefficient between dietary Mg intake and plasma level is 0.13. This is in agreement with other investigators who reported “weak positive correlations between estimated intake and serum Mg level (r = 0.15)” [31]. Notwithstanding these complexities, we adjusted the models for plasma Mg, incident diabetes, and mortality for serum creatinine to minimize the effect of renal function. Consequently, inverse associations with all-cause and CVD mortality became apparent. In order to consider further the impact of impaired renal function on the Mg-mortality associations, we undertook sensitivity analyses by exclusion of those with serum creatinine > 1.5 mg/dL, or those known to have renal disease, and retained the adjustment for serum creatinine. The trend in relationships between plasma Mg and mortality were not altered, although with renal disease, the point estimates were borderline significant. For the joint effect, there were also similar findings with these exclusions (data not shown).

The ionic hypothesis of aging draws attention to the disordered homeostasis of cytosolic calcium and Mg [7]. In support of this hypothesis, experimental dietary Mg deficiency enhances free radical production in skeletal muscle [32]. Population-representative American adults with less than 50% of the recommended daily allowance (RDA) of Mg intake (310–420 mg/day) have CRP values 1.75 times higher than those with at least the RDA for Mg intake [33]. However, we did not find an association between Mg intake and CRP.

### 4.4. Joint Effect

Dietary quality and diversity are inversely associated with mortality [12]. For all-cause mortality, we found that the beneficial effect of dietary and plasma Mg may be dependent on dietary diversity. In our study, those with the highest Mg intakes were more likely to consume whole grains, dairy products, and fruits with related higher nutrient intake profiles for dietary fiber, potassium, and calcium and less fat intake. Some foods and nutrients are themselves associated with a low risk of mortality or chronic diseases, as demonstrated in other studies, but may also be synergistic with Mg in determining these outcomes [34]. Diet and nutrient interactions are complex. From a public-health perspective, our findings support that optimal Mg intake may be most effectively obtained from food rather than supplements, which can lower all-cause mortality risks in older adults. Our data have not provided insight into how particular food patterns optimize Mg status or its biomedical effects. Moreover, the favorable findings for all-cause rather than disease-specific mortality may belie the mechanistic complexity, which might allow the sum of disease-specific events to be realized in total but not individual mortalities. Furthermore, when we excluded deaths in the first year, the joint effect of Mg intake and DDS on all-cause mortality, for the lowest Mg intake group (Q1) and DDS > 4, became significantly protective with an HR (95% CI) of 0.49 (0.26–0.95). This suggests that there are features of dietary diversity that can compensate for relatively low Mg intakes related to survival. 

### 4.5. Sensitivity Analyses

We made several sensitivity analyses (data not shown). By and large these made little or no change to our findings. This applies to exclusion of first year cases and poor renal function, and dietary factors.

For chronic diseases, we have further adjusted for a history of hypertension, stroke, cancer or hyperlipidemia at baseline. The findings were no longer significant. It is most likely that this represents pathways by which Mg deficiency may increase mortality. This would apply particularly to hypertension and stroke [35,36,37]. It is noteworthy that Mg and potassium deficiencies are inter-connected and, therefore, the mediation of the one may be a surrogate for the mediation of the other. Thus, if anything, the loss of significance with the adjustments may underscore the relationships between Mg status and the outcomes we have considered [36].

Smoking is generally a strong confounder in mortality analysis and, in our study, Mg intakes were closely related with smoking status (Table 1). This is likely for several reasons including the displacement of nutritious foods from diet due to an oral habit, a reduction in taste with smoking, and increased metabolic rate satisfied by energy-dense and low nutrient-density foods [38]. As a consequence, smoking itself may serve as a surrogate for Mg intake. We find on the sensitivity analysis that the significance of the association between the joint effect of Mg intakes and DDS on all-cause mortality is lost. Likewise, for plasma Mg, the significances of the associations in all-cause and CVD mortalities, as well as joint effect with DDS, were weakened or lost, even though there were no differences in plasma Mg between smokers and non-smokers. That said, given the homeostatic control of divalent cations like Mg, the total body Mg is likely to be deficient in smokers with important consequences for cellular function relevant to mortality.

### 4.6. Strengths and Limitations

The strengths of our study included the nationally representative sample and the long-term follow-up of a community-based elderly cohort. The dietary and plasma Mg data were used to reflect Mg status. However, our study had limitations. First, the dietary Mg intake assessment was obtained using 24h dietary recall. This does not necessarily reflect long-term food consumption. Given the random error of 24h recall, we cannot avoid the possibility of false negatives in our findings in regard to Mg intake and DM incidence or mortalities. Nevertheless, we have used the information from an SFFQ that enquired about the last month to cross-validate Mg intake by 24h recall (Table 1). This revealed dose-response relationships for several food categories rich in Mg over the Mg intake from a 24h recall. Moreover, older adults are known to have relatively monotonous dietary patterns over a longer period of time [12,39]. We have taken the opportunity to confirm the findings with 24h dietary recall by using Mg intakes estimated from the SFFQ data. The results were materially the same. Second, we did not obtain Mg supplement information and were unable to explore the effect of total Mg intake. Third, all information was collected at the same time at baseline for the cohort study, which limited our ability to make causal inferences. 

### 4.7. Clinical Implications

When lower dietary Mg intakes or plasma Mg concentrations are found in patients, attention should be paid to the quality of diet as a factor in risk evaluation and management. In this population, the two food categories with potential to increase plasma Mg were vegetables and eggs (Table 3), so that these could be emphasized in efforts to improve Mg status. Assessment of dietary diversity is a simple clinical approach for addressing this situation [40]. Mg supplementation may be useful where there is a clear deficiency, such as that which is medication-induced with diuretics or proton pump inhibitors [41], and with identifiable malabsorption or loss.

## 5. Conclusions

Our findings suggest that lower plasma Mg increases all-cause mortality risk in older adults. Dietary quality, judged by its diversity, is interactive with dietary and plasma Mg regarding mortality risk. Even with a single nutrient such as Mg, survival benefits accrue through the consumption of a diverse diet.

## Appendix

**Table A1:** Top five cause-specific mortalities of the study population.

Order	ICD-9 Code	Cause-Specific Mortality	*N*	%
1	140–239	Neoplasms	136	30.33
2	390–459	Diseases of the circulatory system	124	23.6
3	460–519	Acute respiratory infections	66	14.3
4	780–799	Symptoms, signs, and ill-defined conditions	32	8.33
5	580–629	Diseases of the genitourinary system	19	5.36
